# Crosstalk between the Androgen Receptor and PPAR Gamma Signaling Pathways in the Prostate

**DOI:** 10.1155/2017/9456020

**Published:** 2017-10-18

**Authors:** Emuejevoke Olokpa, Patrice E. Moss, LaMonica V. Stewart

**Affiliations:** Department of Biochemistry and Cancer Biology, Meharry Medical College, Nashville, TN 37208, USA

## Abstract

Nuclear receptors are a superfamily of ligand-activated transcription factors that play critical roles in the regulation of normal biological processes and several disease states. Of the nuclear receptors expressed within the prostate, the androgen receptor (AR) promotes the differentiation of prostatic epithelial cells and stimulates production of enzymes needed for liquefaction of semen. Multiple forms of AR also promote the growth of both early and late stage prostate cancers. As a result, drugs that target the AR signaling pathway are routinely used to treat patients with advanced forms of prostate cancer. Data also suggest that a second member of the nuclear receptor superfamily, the peroxisome proliferator activated receptor gamma (PPAR*γ*), is a tumor suppressor that regulates growth of normal prostate and prostate cancers. Recent studies indicate there is a bidirectional interaction between AR and PPAR*γ*, with each receptor influencing the expression and/or activity of the other within prostatic tissues. In this review, we examine how AR and PPAR*γ* each regulate the growth and development of normal prostatic epithelial cells and prostate cancers. We also discuss interactions between the AR and PPAR*γ* signaling pathways and how those interactions may influence prostate biology.

## 1. Introduction

The prostate is an integral part of the male reproductive system. This walnut sized organ is located directly below the bladder and in front of the rectum, above the muscles of the pelvic floor. The prostate manufactures a fluid that contains large amounts of prostate-specific antigen (PSA), a kallikrein-like serine protease that degrades proteins to promote liquefaction of semen [1–3]. It also contains epithelium-lined glands as well as a muscular component which helps to push semen into the urethra so that it can be expelled from the body during ejaculation.

One of the primary diseases that affect the prostate is prostate cancer. When one considers only nonskin cancers, prostate cancer is the most common cancer detected within American men. According to the American Cancer Society, there will be 161,360 newly diagnosed cases of prostate cancer in the United States in 2017 [4]. In addition, it is estimated that 26,730 men will die of this disease this year [4]. Although prostate cancer is currently the third most common cause of cancer death in US males, the actual death rates for prostate cancer have decreased since the early 2000s. During this time period the incidence of prostate cancer has also decreased [4]. This reduction is believed to be due in part to recent recommendations that have advised against the use of routine PSA screening as a strategy for early detection of prostate cancer. There are several key risk factors that have been associated with the development of prostate cancer. These include having a family history of the disease, increasing age, and ethnicity. Worldwide the incidence of prostate cancer is highest in African American men in the United States and Caribbean men of African descent. Furthermore, in the United Stated the number of prostate cancer associated deaths is higher in African American men than in Caucasian American men. Inherited genetic conditions have also been associated with prostate cancer, for an elevated risk of prostate cancer has been noted in men with Lynch syndrome and men that carry* BRCA1* and* BRCA2* gene mutations [5, 6]. In addition, recent studies suggest that obesity may increase one's risk for aggressive forms of prostate cancer [7–9]. Prostate cancer originates from epithelial cells within the prostatic glands and can metastasize to the regional lymph nodes, bone, and distant organs such as liver, lung, and brain. While elevated PSA levels may suggest a patient has prostate cancer, needle biopsies are the primary diagnostic tool used to confirm the presence of this disease. Primary treatment options for patients with prostate cancer vary based on the age of the patient, tumor grade, and tumor stage. According to the National Comprehensive Cancer Network (NCCN) [10], observation, active surveillance, radiation, and radical prostatectomy are possible therapies for patients with very low (TMN Stage T1c, N0, M0; PSA < 10 ng/mL; Gleason grade ≤ 6) or low risk (TMN Stage T1a, T1b, or T2a, N0, M0; PSA < 10 ng/mL; Gleason grade ≤ 6) prostate cancer. Observation is usually reserved for patients with a limited life expectancy (<10 years), while active surveillance, radiation, and radical prostatectomy are reserved for patients that are expected to live at least 10 years or longer. Patients diagnosed with intermediate risk prostate cancer (TMN Stage T2b or T2c, N0, M0; PSA 10–20 ng/mL; or Gleason grade 7) with a limited life expectancy can be treated with observation or radiation, while those expected to live greater than 10 years are often treated with radical prostatectomy or radiation. Patients with high risk cancer (TMN Stage T3a, N0, M0; PSA > 20 ng/mL; or Gleason grade 8–10) are often treated with radical prostatectomy or radiation in combination with androgen deprivation therapy (ADT). ADT is also a standard therapy for patients who have prostate cancer that has spread to the regional lymph nodes or metastasized to distant sites. If prostate cancer is detected and treated when it is primarily localized to the prostate, the chances of patient survival are high. However, once prostate cancer metastasizes to sites outside the prostate the number of deaths associated with this disease dramatically increases. This is reflected in the fact that the five-year relative survival rate in the United States for patients with localized or regional prostate cancer during the period 2006–2012 was approximately 100%, while the five-year relative survival rate for metastatic prostate cancer was 29% [4].

The nuclear receptor superfamily consists of receptors that play critical roles not only in normal prostate development but also in prostate cancer. Two members of the nuclear receptor superfamily that regulate prostate growth and differentiation are the androgen receptor (AR) [11] and the peroxisome proliferator activated receptor gamma (PPAR*γ*). Both AR and PPAR*γ* are ligand-activated transcription factors that bind to hormone response elements in DNA and regulate transcription in order to control expression of gene products that regulate multiple cellular processes, such as cell proliferation, cell survival, and metabolism. AR binds and mediates the biological effects of androgens such as testosterone and dihydrotestosterone (DHT) [12]. Ligands for PPAR*γ* include polyunsaturated fatty acids and derivatives of arachidonic acid as well as synthetic compounds. PPAR*γ* was initially identified as a receptor that regulates differentiation of adipocytes. However, PPAR*γ* is also expressed within the prostate and appears to influence the activity of prostatic cells. This review will discuss our current understanding of the role of AR and PPAR*γ* within the prostate and how interactions between these two signaling pathways can influence the growth and development of normal prostate as well as prostate cancer.

## 2. AR in Normal and Diseased Prostate

Multiple forms of AR have been detected within humans. The first AR cDNA sequence to be characterized was initially reported in 1988 [13, 14]. This version of AR, which is often referred to as full-length AR (AR-FL), encodes a 110 kD protein that contains all four of the major functional domains present in nuclear receptors: a C-terminal ligand binding domain (LBD), a hinge domain, the DNA binding domain (DBD), and the N terminal domain (NTD) [15]. In the absence of androgens, AR-FL resides primarily in the cytoplasm as part of a large multiprotein complex that contains heat shock proteins [16]. Ligand binding induces dissociation of AR-FL from this complex, homodimerization, and rapid translocation of the receptor to the nucleus [17, 18]. Within the nucleus, AR-FL regulates the transcription of AR target genes by binding to androgen response elements (AREs) [18].

The AR-FL is expressed in many tissues including the prostate and testes [19]. Within the prostate AR plays a key role in two major cell types, mesenchymal cells and epithelial cells. The mesenchyme, or stroma, of the prostate consists mainly of smooth muscle cells and fibroblasts [20–23]. The stroma surrounds the glands within the prostate and is responsible for producing many of the growth factors that regulate the growth and development of prostatic epithelial cells [21, 24, 25]. The prostate epithelial cells form glands within the prostate and include luminal/secretory cells, basal cells, and neuroendocrine cells [26]. The luminal cells, which line prostatic glands, are responsible for the production of PSA and other secreted proteins that assist with liquefaction of semen. AR-FL has been detected in prostatic stroma and the luminal epithelial cells. However, the levels of AR-FL within each cell type vary during one's lifespan. In the fetus the prostate develops from the urogenital sinus, which consists of an inner layer of urogenital sinus epithelium (UGE) and an outer layer of urogenital sinus mesenchyme (UGM). During fetal development AR-FL is undetectable within the UGE but is present within the UGM. AR-mediated signaling within the UGM stimulates UGE growth and differentiation, for AR-negative UGM is unable to induce prostate formation [27]. Furthermore, AR knockdown within the fibroblast and/or smooth muscle of the stroma suppresses prostate epithelium growth and development [28, 29]. These data suggest stromal AR is needed for proper prostate development. In the normal adult prostate, significant amounts of AR-FL are present in the stromal cells and luminal epithelial cells [30, 31]. Low levels of AR are also present in basal epithelial cells [30]. However, basal epithelial cells do not require androgen for their growth [32]. Androgens can stimulate prostate growth and differentiation directly via AR-FL activation within the epithelial cells and indirectly via stromal AR [33, 34]. Stromal AR signaling functions to modulate different growth factors, including fibroblast growth factors (FGFs) [35, 36], insulin-like growth factor I (IGF-I) [37], and vascular endothelial growth factor (VEGF) [36]. These AR-induced growth factors then diffuse through the stroma and act in a paracrine fashion on epithelial cells to facilitate prostate growth and differentiation [23, 29, 38–40]. While each of these growth factors is crucial for prostate development, no single growth factor is able to completely replace AR signaling. AR-FL plays a critical role not only in normal prostate but also in prostate cancer. AR-FL continues to be expressed in the cancer cells that arise from prostatic epithelium as well as some cancer associated stromal cells. AR-FL within the epithelial derived tumor cells promotes growth of early stage and advanced prostate tumors. Stromal AR also promotes prostate carcinogenesis, for AR-positive stromal cells stimulate tumor development in tissue recombinant studies involving RWPE-1 cells that lack AR as well as AR-negative BPH-1 prostatic cells [41, 42]. While AR continues to be expressed in many advanced prostate cancers, AR expression is lost in stromal cells during the process of tumor progression and the development of metastatic tumors. Reductions in tumor associated stromal AR-FL have been linked to poor patient outcome as well as disease progression [43]. The reason underlying the loss of stromal AR with prostate cancer progression is unclear. However, clonal selection of AR-negative cells, altered expression of paracrine factors the regulate AR expression, mutations within the AR gene and epigenetic silencing of AR are some of the factors that may contribute to this net decrease in stromal AR-FL levels [43].

In addition to the 110 kD AR-FL, shorter AR variants (ARVs) have been observed in humans. Most of these ARVs retain the NTD and DBD but lack the LBD, making them constitutively active [44]. ARVs, which normally reside in the nucleus of the cell, are believed to arise primarily from alternative splicing of AR transcripts. However, AR gene mutations and rearrangements as well as proteolytic cleavage of AR-FL have also been proposed as a mechanism for AR variant generation [45–47]. Analysis of the human prostate cancer xenografts LuCAP86.2 and LuCaP136, immortalized prostate cell lines (namely, 22Rv1, VCaP, and CWR-R1), and tumor samples from prostate cancer patients has led to the identification of seventeen ARVs [47–52]. It is important to note that mRNAs for only ten of the seventeen identified ARVs (i.e., ARQ640X, AR-V1, AR-V5, AR-V6, AR-V7, AR-V9, ARV^567es^, AR13, AR-V14, and AR8) have been detected in tumor samples from patients with castration-resistant prostate cancer. Some ARVs also appear to be expressed in normal prostate tissue and benign prostatic hyperplasia. However, in these tissues the relative amount of ARV mRNA and/or protein expression is lower than that of AR-FL [47]. In prostate cancers, ARV expression is generally higher in castration-resistant prostate cancers than in less aggressive, androgen-dependent prostate tumors. It is believed this increased ARV expression in castration-resistant tumors is an adaptive response to androgen deprivation therapy and provides a survival advantage to prostate cancer cells. AR-V7 appears to be one of the major AR variants present in human castration-resistant prostate tumors and prostate cancer cell lines [50, 53, 54]. High expression of AR-V7 is associated with the development of castration-resistance, tumor recurrence, and prostate cancer survival [48, 50, 53]. Elevated AR-V7 levels have also been detected in circulating prostate tumor cells isolated from patients that are resistant to enzalutamide and abiraterone acetate, two relatively new drugs that target the AR signaling pathway [55, 56]. Furthermore, selective knockdown of AR-V7 in the castration-resistant CWR-R1 and 22Rv1 prostate cancer cell lines significantly inhibited growth of cells in media depleted of androgens [50]. Recent work by Kohli et al. suggests that a second variant, AR-V9, is coexpressed with AR-V7 in castration-resistant prostate cancers. Further analysis of AR-V9 in this study revealed that AR-V9 expression promotes androgen-independent growth of prostate cancer cell lines and is linked to abiraterone resistance [57]. It has been suggested that ARVs must work in concert with AR-FL to regulate expression of AR target genes and stimulate growth of castration-resistant cancers [52]. However, there are reports that two major ARVs, AR-V7 and ARV^567es^, not only heterodimerize with AR-FL but also form homodimers [58]. Therefore, ARVs may be able to regulate gene expression independently of AR-FL. In addition to modulating expression of standard AR target genes such as PSA, AR-V7 and ARV^567es^ appear to regulate unique target gene sets that are distinct from those regulated by AR-FL [50, 59, 60].

In early stage prostate cancer, the ligand-activated AR-FL appears to be the form of AR that primarily regulates tumor growth. Huggins and Hodges discovered in 1941 that androgens, the ligands that activate AR-FL, play a vital role in prostate cancer [61]. These authors showed that androgen deprivation therapy (ADT), which decreases circulating androgen levels, suppresses tumor progression in most cases [62]. This ground-breaking discovery coupled with the fact that approximately 70–80% of patients respond to ADT therapy has made ADT the standard therapy to treat metastatic prostate cancer. While ADT is initially effective in reducing the prostate tumor burden, this response is usually not long lived. Many men develop recurrent, castration-resistant forms of prostate cancer approximately 18–24 months after they begin ADT [63]. Currently men with castration-resistant prostate cancer show a poor prognosis. However, the drugs that interfere with the AR signaling pathway remain a viable treatment option for patients with these aggressive tumors. As noted in multiple review articles, AR activation in castration-resistant prostate cancers appears to occur via at least six potential mechanisms [11, 64–66]. Overexpression of AR-FL protein can occur as a result of AR gene amplification within tumor tissues, while single AR gene point mutations generate promiscuous forms of AR that can be activated by a wider range of ligands. Under castrate conditions AR-FL can be activated via intratumoral production of androgens and adrenal androgens. Increased expression of AR cofactor/coregulator proteins as well as ligand-independent activation of AR via growth factors and cytokines can also enhance AR signaling within castration-resistant prostate cancer cells. Finally, expression of ARVs within these tumors may allow for the ligand-independent transcription of AR target genes. The presence of AR-FL and ARVs protein and/or mRNA has been detected in castration-resistant prostate cancer cells [48, 50, 52–54, 60]. The Federal Drug Administration has recently approved two novel agents, abiraterone acetate and enzalutamide, as treatments for metastatic, castration-resistant prostate cancer. While both compounds improve the survival of patients with castration-resistant prostate cancer, they interfere with AR signaling via different mechanisms. Abiraterone acetate reduces AR activation by inhibiting the synthesis of intratumoral and extratumoral androgens while enzalutamide functions as a potent AR antagonist. Unfortunately not all patients respond to these therapeutic agents, and the development of drug resistance remains an issue for both drugs (reviewed in [67, 68]). This may be especially true for tumors that express high levels of the constitutively active AR-V7 and other AR variants, for it is believed that ARV expression contributes to drug resistance within prostate cancers. As a result there is still a desire to identify novel treatments for prostate cancer that interfere with AR signaling, especially those compounds that target AR regions outside of the LBD [69].

## 3. PPAR**γ** in Normal and Diseased Prostate

PPAR*γ* is a ligand-activated transcription factor that has a domain structure similar to that of AR and other members of the nuclear hormone receptor superfamily. It possesses a highly conserved DBD, a ligand-independent activation domain in the NTD, a hinge region, and a LBD. The unliganded form of PPAR*γ* normally resides in the cell nucleus bound to regions of DNA known as PPAR response elements (PPREs). In this inactive state, PPAR*γ* is associated not only with the retinoid X receptor (RXR) but also with corepressor proteins that suppress the receptor's transcriptional activity. Ligand binding induces disassociation of corepressors from the PPAR*γ*/RXR heterodimer complex and recruits proteins such as RNA polymerase II and the coactivator proteins steroid receptor coactivator-1 (SRC-1) and peroxisome proliferator activated receptor coactivator 1 alpha (PGC-1*α*) to modulate transcription of PPAR*γ* target genes within the prostate [70]. Phosphorylation sites located within the NTD region at Serine 82 and the hinge region at Serine 273 negatively regulate PPAR*γ* function [71]. PPAR*γ* can also be sumoylated within its NTD at Lysine 33 and Lysine 77 and LBD at Lysine 367. Sumoylation of PPAR*γ* at Lysine 33 and Lysine 77 appears to inhibit PPAR*γ*'s ability to induce gene expression, while sumoylation within the LBD at Lysine 367 is required ligand-induced transcriptional repression by this receptor [72–74]. In humans, the PPAR*γ* gene is located on chromosome 3 (specifically 3p25.2) and gives rise to two major PPAR*γ* isoforms (PPAR*γ*1 and PPAR*γ*2) by alternative splicing [75–77]. The expression of each isoform varies throughout the body. PPAR*γ* isoforms are expressed in prostatic epithelial cells from normal tissue and prostate cancers [78, 79]. Furthermore, data suggest PPAR*γ*1 is the predominant isoform present in prostate tumors [79].

In the normal prostate, PPAR*γ* appears to play a critical role in growth and differentiation. Jiang et al. demonstrated that conditional knockout of PPAR*γ* within mouse prostatic epithelial cells results in the development of low grade prostatic intraepithelial neoplasia (PIN), a lesion which is believed to be a precursor of prostate cancer [71, 80]. Furthermore, this group found that the development of mouse PIN in PPAR*γ* knockout mice was associated with decreased differentiation of the secretory luminal epithelium as well as an increase in autophagy and oxidative stress [80]. Thus, PPAR*γ* appears to function as a tumor suppressor that controls cell survival and differentiation within normal prostate epithelium. However, in certain contexts the presence of PPAR*γ* may actually promote tumor growth. Ahmad et al. used a transposon-based “sleeping beauty” screen to identify additional genes that can enhance the development of prostate tumors. In their study, increases in PPAR*γ* expression coupled with loss of the PTEN tumor suppressor enhanced prostate tumorigenesis [81]. Therefore, the role of PPAR*γ* in prostate cancer development may vary depending on the expression levels of other tumor suppressors and proteins that control tumor growth.

Both PPAR*γ* mRNA and protein have been detected within human prostate tumor tissue sections and prostate cancer cell lines. However, conflicting data exist regarding the relative expression of PPAR*γ* in normal/benign prostate versus prostate cancers. An analysis of normal and prostatic adenocarcinomas performed by Mueller et al. in 2000 suggested that normal prostate contains a higher amount of PPAR*γ* than prostate cancers [82]. However, subsequent studies have indicated that human PIN and prostate cancers express more PPAR*γ* mRNA and protein than normal prostate epithelial cells [79, 83]. PPAR*γ* mRNA and protein have been detected in castration-sensitive as well as castration-resistant prostate cancer cell lines (Table 1). However, the level of PPAR*γ* expression within these cell lines does vary. Mueller et al. analyzed PPAR*γ* levels in the castration-sensitive LNCaP cells and two AR-negative, castration-resistant cell lines, DU145 and PC-3 cells. Although PPAR*γ* mRNA was expressed in all of the cell lines, the lowest amount of PPAR*γ* was detected in the LNCaP cells. DU145 contained intermediate PPAR*γ* levels, while PC-3 cells expressed the highest amount of PPAR*γ* mRNA. Western blots revealed the PC-3 cells expressed the highest level of PPAR*γ* protein. Also, a significant amount of the PPAR*γ* protein within PC3 cells appeared to be highly phosphorylated [82]. We used Western blots and luciferase-based transcriptional assays to measure PPAR*γ* expression and activity in LNCaP cells and C4-2 cells, a castration-resistant derivative of the LNCaP cell line, in order to determine whether PPAR*γ* levels change as tumors become castration-resistant. Our results indicated that castration-resistant C4-2 cell line contains a greater amount of functional PPAR*γ* than the castration-sensitive LNCaP cells [84]. These results complement data from http://www.cbioportal.org that demonstrates the PPAR*γ* gene is amplified in approximately 27% of patients with castration-resistant prostate cancer [81]. Together, these studies suggest that one change that accompanies tumor progression to castration-resistance is an increase in the amount of functional PPAR*γ*.

Compounds that have been identified as ligands for PPAR*γ* appear to use PPAR*γ*-dependent as well as PPAR*γ*-independent signaling pathways to regulate prostate cancer growth. Both naturally occurring and synthetic PPAR*γ* ligands decrease prostate cancer cell proliferation. The endogenous PPAR*γ* ligands 15-deoxy-Δ^12,14^-prostaglandin J2 (15dPGJ_2_) and 15(S)-hydroxyeicosatetraenoic acid (15S-HETE) reduce proliferation and inhibit cell cycle progression in LNCaP, PC-3, and/or DU145 cells [78, 85–87]. 15S-HETE also reduced the ability of the growth factors EGF and IGF-I to stimulate phosphorylation of Erk MAP kinase in PC-3 cells [85]. The antiproliferative effects of 15S-HETE appear to require activation of PPAR*γ*, for concentrations of 15S-HETE that suppressed PC-3 cell proliferation were also effective in inducing expression of PPAR*γ* target genes [86]. However, it was demonstrated by Chaffer et al. that the PPAR*γ* antagonist GW9662 did not alter the ability of 15dPGJ_2_ to reduce prostate cancer cell proliferation. It therefore appears that 15dPGJ_2_ regulates proliferation via a PPAR*γ*-independent pathway [78]. Synthetic PPAR*γ* ligands include a class of drugs known as the thiazolidinediones (TZDs). The TZDs troglitazone, rosiglitazone, ciglitazone, and pioglitazone decrease* in vitro* and* in vivo* proliferation of castration-sensitive (LNCaP) and castration-resistant (C4-2, DU145, and PC-3) human prostate cancer cell lines [71, 78, 88–91]. TZDs also inhibit proliferation of human prostate cancer cells grown as primary cultures [92]. In addition, the TZD pioglitazone has been shown to reduce proliferation and prostate cancer progression within the transgenic rat for adenocarcinoma of prostate (TRAP) model of prostate cancer [93]. The antiproliferative effects of TZDs have been associated with cell cycle arrest as well as the induction of apoptosis. These changes have been linked to TZDs-induced regulation of proteins that play key roles in critical signaling pathways, such as c-Myc, NF*κ*B, cyclin D1, and GSK-3*β* [84, 88, 94–102]. Furthermore, we and others have demonstrated TZD-induced alterations in protein expression, cell cycle progression, and apoptosis can occur via PPAR*γ*-dependent and PPAR*γ*-independent signaling pathways [78, 82, 88–90].

## 4. PPAR**γ** Effects on AR Signaling

Studies performed by Strand et al. have provided the most information regarding the regulation of AR signaling by PPAR*γ* isoforms within the normal prostate. In these experiments, the authors used prostatic epithelial cell lines to examine the effect of each PPAR*γ* isoform on AR activity. Their results revealed that stable transfection of PPAR*γ*1 into mouse prostatic epithelial cells lacking PPAR*γ* (mPrE-*γ*KO cells) reduced AR transcriptional activity. However, restoration of PPAR*γ*2 to mPrE-*γ*KO cells increased activation of AR by the ligand DHT [103]. Therefore, PPAR*γ* may influence the function of AR in normal prostatic epithelial cells in an isoform-specific manner.

**Figure 1:**
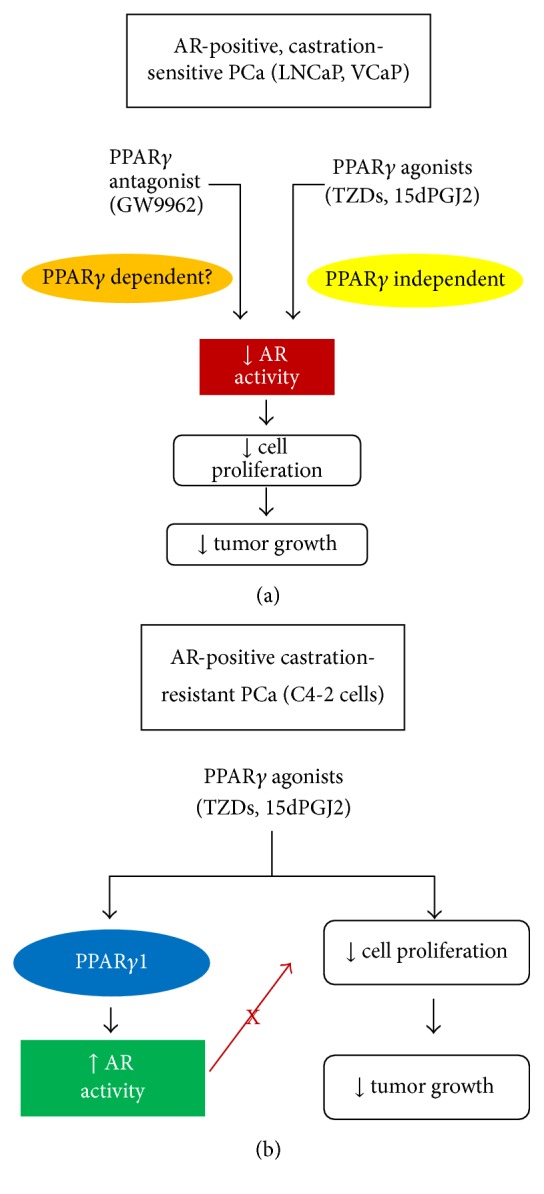
PPAR*γ* ligands regulate AR signaling within human prostate cancer cells. In AR-positive castration-sensitive prostate cancers such as LNCaP and VCaP cells (a), PPAR*γ* agonists and the PPAR*γ* antagonist GW9662 have been shown to decrease AR transcriptional activity. Agonist-induced reductions in AR signaling appear to occur independently of PPAR*γ*, while it is currently not known if PPAR*γ* is required for GW9662-induced reductions in AR activity. It is believed that these reductions in AR signaling contribute to the antiproliferative and antitumor effects of PPAR*γ* ligands in these early stage cancers. Conversely, PPAR*γ* agonists appear to enhance AR signaling via a PPAR*γ*1-dependent mechanism in the AR-positive, castration-resistant C4-2 cells (b). Data suggest that the antiproliferative effects of PPAR*γ* agonists in AR-positive cells do not require PPAR*γ* and appear to occur independently of any increases in AR activity within these cells. However, since AR activation can drive growth of castration-resistant tumors, it is possible PPAR*γ* agonist-induced increases in AR activity may interfere with the net ability of these compounds to reduce cell proliferation and tumor growth in AR-positive, castration-resistant cancers.

Multiple reports have shown that treatment of human prostate cancer cells with PPAR*γ* ligands alters AR signaling. However, the effect of PPAR*γ* ligands on AR activity appears to vary between AR-positive, castration-sensitive and AR-positive, castration-resistant human prostate cancer cells. Hisatake et al. performed luciferase-based reporter assays in the castration-sensitive, AR-FL positive LNCaP cells to measure the effect of TZDs on AR transactivation. The results from these experiments showed TZDs pioglitazone and troglitazone significantly reduced ligand-induced activation of AR in the LNCaP cell line. In their study troglitazone also inhibited DHT-induced increases in PSA protein, which is directly regulated by AR [104]. In a separate study Yang et al. noted troglitazone reduces ligand-induced activation of AR in LNCaP cells. This group also demonstrated troglitazone decreased both basal and DHT-dependent PSA protein expression [105]. Within LNCaP cells, these troglitazone-stimulated reductions in AR expression and transcriptional activity did not require activation of PPAR*γ*. Instead, decreases in AR signaling that were stimulated by high micromolar concentrations of troglitazone (≥40 *μ*M) appeared to be mediated by proteasomal degradation of the transcription factor Sp1 [106]. Studies performed by our research group revealed that the TZD ciglitazone produced varying effects on AR signaling in the castration-sensitive LNCaP cells and C4-2 cells, an AR-positive, castration-resistant derivative of the LNCaP cell line. Our data indicated that ciglitazone uses a PPAR*γ* dependent mechanism to stimulate AR activation in C4-2 cells. However, ciglitazone inhibits AR-mediated transcription and the expression of AR target genes in LNCaP cells via a PPAR*γ*-independent signaling pathway [84]. Although we did not detect a reduction in AR signaling in C4-2 cells exposed to ciglitazone, ciglitazone did inhibit proliferation of the C4-2 cell line. Therefore, the regulation of AR by ciglitazone (and possibly other TZDs) in AR-positive, castration-resistant prostate cancer cells may not prevent ciglitazone-induced reductions in cell proliferation (Figure 1). To date, published reports have described the effects of endogenous PPAR*γ* ligands on AR signaling only within AR-positive, castration-sensitive prostate cancer cells. One commonly used endogenous PPAR*γ* ligand, 15dPGJ_2_, does reduce AR activation in LNCaP [104, 107] and VCaP [108] prostate cancer cells. Work by Kaikkonen et al. suggests that the ability of 15dPGJ_2_ to reduce AR signaling is not associated with PPAR*γ* activation. Instead, it appears 15dPGJ_2_ blocks AR function by forming adducts with AR, inducing AR sumoylation, and disrupting AR structure [108]. A recent study has suggested that the PPAR*γ* antagonist GW9662 also regulates AR signaling within AR-positive, castration-sensitive prostate cancers. Work done by Tew et al. revealed that GW9662 reduced DHT-mediated increases in PSA-luciferase activity in LNCaP cells [109]. Although it is well accepted that GW9662 functions as an irreversible PPAR*γ* inhibitor, GW9662 has also been reported to regulate cell proliferation via a PPAR*γ*-independent pathway [110]. At present it is unclear whether GW9662-mediated suppression of AR occurs via a PPAR*γ*-dependent or PPAR*γ*-independent pathway. However, these data do provide additional evidence that multiple types of PPAR*γ* ligands can modulate AR signaling within prostate cancer cells.

## 5. AR Effects on PPAR**γ** Signaling

**Figure 2:**
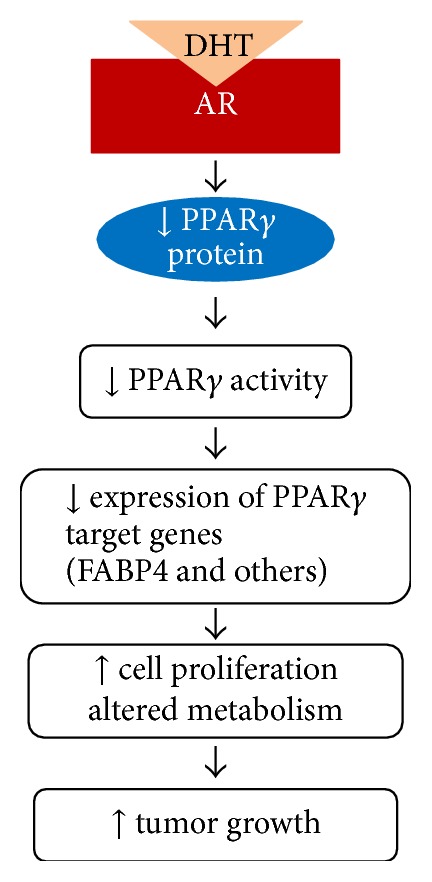
AR regulates PPAR*γ* signaling in human prostate cancers. Data from our laboratory suggest that androgen-induced AR activation produces a net decrease in PPAR*γ* protein and PPAR*γ* activity in AR-positive castration-sensitive (VCaP) and castration-resistant (C4-2) prostate cancer cells. These decreases in PPAR*γ* transcriptional activity result in reduced expression of FABP4 and other PPAR*γ* target genes and ultimately alterations in cancer cell proliferation and metabolism that promote tumor growth.

To date there are no published studies that have directly examined the regulation of PPAR*γ* by the AR signaling pathway in normal prostatic tissues. However, recent data from our laboratory suggest that AR activation can suppress PPAR*γ* expression and/or activity within human prostate cancers. The AR agonist DHT at nanomolar concentrations produced a significant decrease in PPAR*γ* mRNA and protein levels within two AR-positive human prostate cancer cell lines: the castration-sensitive VCaP cells and castration-resistant C4-2 cell line (Figure 2). In addition, PPAR*γ* expression and activity within C4-2 cells were increased following siRNA-mediated knockdown of AR-FL or exposure to AR antagonists [111]. We also explored the effect of AR-FL on PPAR*γ* expression and function within PC-3 cells, which lack AR but express a significant amount of PPAR*γ*. Overexpression of AR-FL in PC-3 cells did not dramatically alter PPAR*γ* protein levels. However, increasing AR-FL levels within the PC-3 cells did inhibit ligand-stimulated PPAR*γ* transcriptional activity [111]. Taken together, these data indicate that AR-driven reductions in PPAR*γ* activity can occur independently of reductions in PPAR*γ* protein levels. The human prostate cancer cell lines that were used in our study predominantly express PPAR*γ*1. However, Singh et al. showed that androgens suppress PPAR*γ*2 expression in mouse adipocytes [112]. Therefore androgens have the potential to regulate expression of both PPAR*γ* isoforms within normal prostate and prostate cancers.

The functional consequences of AR-stimulated decreases in PPAR*γ* expression and activity are not fully understood. Our data indicate that, at a minimum, these reductions affect PPAR*γ*'s ability to regulate gene expression in cancer cells. Direct target genes for PPAR*γ* include gene products that play key roles in fatty acid transport and metabolism such as lipoprotein lipase [113] and adipocyte fatty acid binding protein (FABP4) [70]. Of the fatty acid binding proteins (FABPs) present in mammalian tissues, FABP4 and cutaneous FABP (FABP5) have been extensively studied in prostate cancer. FABP5, whose expression is directly regulated by PPAR*β*/*δ*, binds fatty acids that serve as ligands for PPAR*γ* and enhances prostate cancer cell proliferation [114]. FABP4 is a protein directly regulated by PPAR*γ* that has also been linked to prostate cell proliferation and survival. Data from De Santis et al. revealed that apoptosis could be induced in DU-145 prostate cancer cells via overexpression of FABP4 [115]. To define the effect of AR on PPAR*γ* function, our lab explored how changing AR expression within prostate cancer cells influenced PPAR*γ*-induced increases in FABP4. We found that overexpression of AR within PC-3 cells inhibits PPAR*γ*-mediated increases in FABP4 mRNA within PC-3 cells [111]. Our studies also revealed that reducing AR-FL expression by siRNA-mediated knockdown enhanced the ability of rosiglitazone to suppress proliferation of C4-2 prostate cancer cells [111]. Together, these results indicate AR may enhance prostate cancer growth and survival by suppressing the induction of FABP4 by PPAR*γ*. AR also functions as a regulator of metabolism within human prostate cancer cells. Work by White et al. has shown that androgens promote glutamine uptake in AR-positive prostate cancer cell lines by inducing expression of two glutamine transporters, SLC1A4 and SLC1A5 [116]. Furthermore, ChIP-seq analysis performed using RNA from the AR-positive LNCaP and VCaP prostate cancer cell lines indicates AR directly regulates expression of glucose transporter 1 (GLUT1), hexokinases I and II, phosphofructokinase, CAMKK2, mTOR, and other gene products that play key roles in lipid and glucose metabolism [117]. It is possible that AR uses at least two different strategies to control metabolism in prostate cancers cells: the direct regulation of AR target genes and modulation of PPAR*γ* function. However, additional studies must be performed to clarify the extent to which reductions in PPAR*γ* function contribute to AR's ability to regulate prostate cancer proliferation and metabolism.

## 6. Factors That Influence AR-PPAR**γ** Crosstalk

### 6.1. Nuclear Receptor Coregulators

The transcriptional activity of AR and PPAR*γ* is influenced by the recruitment of coregulator proteins. These include coactivators, which enhance receptor transcriptional activity, and corepressors, which suppress receptor-mediated transcription. The peroxisome proliferator activated receptor coactivator 1 alpha (PGC1*α*) may be one coactivator that modulates interactions between AR and PPAR*γ*. PGC-1*α* was originally identified in 1998 as a protein that interacts with the DBD and hinge region of PPAR*γ* and enhances the ability of PPAR*γ* to induce target gene expression [118]. However, a recent study by Shiota et al. suggests that PGC-1*α* may also function as an AR coactivator. Through luciferase-based reporter assays this group demonstrated that PGC-1*α* increased AR-mediated transcription. Furthermore, they showed siRNA-mediated knockdown of PGC-1*α* reduced LNCaP cell proliferation and lowered expression of the AR target gene PSA [119]. Subsequent work by Tennakoon et al. confirmed that loss of PGC-1*α* reduced proliferation of AR-positive prostate cancer cell lines, specifically LNCaP and VCaP cells. However, in their hands PGC-1*α* did not enhance AR-mediated transcription. Instead they found that androgens used an AMPK-dependent signaling pathway to increase PGC-1*α* levels in prostate cancer cells [120]. PGC-1*α* is expressed in castration-sensitive and castration-resistant prostate cancer cell lines [119, 120]. In addition, it appears that PGC-1*α* levels are elevated in a subset of human prostate tumors [120]. While the exact nature of the interaction between AR and PGC-1*α* needs to be further clarified, it is clear that AR signaling and PGC1*α* are linked. Therefore, AR-PGC-1*α* crosstalk may compromise PPAR*γ* function within prostate cancers. The transcriptional activity of PPAR*γ* and AR is also enhanced by two members of the p160 family of coactivators, SRC-1 and TIF-2 [121–124]. These coactivators may play a greater role in receptor signaling within advanced tumors, for SRC-1 and TIF-2 protein levels are elevated in metastatic and recurrent prostate cancers [125, 126]. It remains to be determined whether SRC-1 and TIF-2 influence interactions between the AR and PPAR*γ* signaling pathways.

Regulation of corepressor levels appears to be one way PPAR*γ* agonists control AR function. Cyclin D1 (CD1) is a corepressor that inhibits ligand-induced activation of AR by physically interacting with the receptor's N terminus region within the nucleus of the cell [127, 128]. This corepressor function of CD1 also occurs independently of CD1's ability to regulate cell cycle progression and does not require a LXXLL motif present within the CD1 protein [127, 128]. We have shown that ciglitazone lowers CD1 protein levels in the castration-resistant C4-2 cells but not in the castration-sensitive LNCaP cell line. This reduction in CD1 appears to be required for ciglitazone to enhance AR signaling in C4-2 cells, for this response is blocked in C4-2 cells that overexpress CD1 [84]. CD1 may not be the only corepressor that influences AR-PPAR*γ* interactions. AR-mediated transcription can also be reduced by a second protein known as nuclear corepressor (NCoR) [129, 130]. NCoR suppresses PPAR*γ* activation within adipocytes by promoting phosphorylation of PPAR*γ* at Ser 273 [131]. NCoR also inhibits PPAR*γ* transcriptional activity and reduces the effects of PPAR*γ* agonists in PC-3 cells [132]. It is not known whether androgens or PPAR*γ* ligands regulate NCoR expression or activity within prostate cancer cells. However, it is possible that activation of either receptor alters the availability of NCoR and other corepressors. This would increase the pool of corepressors that can suppress AR- or PPAR*γ*-mediated transcription and ultimately result in a net decrease in receptor function.

### 6.2. MicroRNAs That Control AR and PPAR*γ* Expression

Crosstalk between the AR and PPAR*γ* signaling pathways can also be influenced by the amount of each receptor present within human prostate cancer cells. One group of molecules that are known to modulate AR and PPAR*γ* protein levels via a posttranscriptional mechanism are microRNAs (miRNAs). miRNAs represent small noncoding RNA that regulate target mRNA expression by suppression of their translation and/or selective cleavage. Due to these activities, miRNAs can modulate protein expression and alter signaling pathways that control cell functions shifting cells toward cell proliferation, differentiation, and/or apoptosis. Multiple miRNAs, such as miR-27a, miR-27b, miR-130, miR-302, and miR-34, have been reported to suppress PPAR*γ* levels [133–137]. Of these, miR-27a is a direct AR target gene. Expression of miR-27a is upregulated by AR agonists in LNCaP and PC3 wt-AR cells [138]. Furthermore, preliminary studies from our lab indicate miR-27a and miR-27b are induced by DHT concentrations that lower PPAR*γ* expression in C4-2 cells (data not shown). Additional studies are required to determine whether DHT-stimulated increases in miR-27a and/or miR-27b contribute to the DHT-mediated reductions in PPAR*γ* mRNA within prostate cancer cell lines. Like PPAR*γ*, AR levels can also be controlled within normal and diseased tissues by miRNAs. Work by Östling et al. suggests over 70 different miRNAs can modulate expression of AR within human prostate cancer cells [139]. At present, it is unknown whether any of these miRNAs are also regulated by PPAR*γ* agonists within prostate cancer cells. However, it is possible that ligand-induced changes in expression of miRNAs that control the net amount of each receptor or its associated coregulators could influence the extent of AR-PPAR*γ* interactions within normal prostate and prostate cancers.

## 7. Conclusions

AR and PPAR*γ* have each been recognized as a receptor that regulates prostate growth and differentiation. Studies performed by our laboratory and others strongly suggest that interactions between the AR and PPAR*γ* signaling pathways may also influence prostate biology. On one hand, PPAR*γ* ligands can either suppress or enhance the transcriptional activity of AR. In the normal prostate, the net effect of PPAR*γ* on AR signaling is determined in part by the PPAR*γ* isoform that is expressed within prostatic epithelial cells. In prostate cancers, the ability of PPAR*γ* to regulate AR function varies depending on the ability of tumors to respond to castration. While PPAR*γ* ligands suppress AR activation in AR-positive, castration-sensitive prostate cancers via mechanisms that are independent of PPAR*γ*, ligand-induced activation of PPAR*γ* increases AR signaling in AR-positive, castration-resistant prostate cancer cells. On the other hand, activation of AR reduces PPAR*γ* levels and activity within human prostate cancers. This suggests that any reduction in AR signaling would increase the amount of functional PPAR*γ* and enhance the antitumor effects of PPAR*γ* within prostate cancers. Interactions between the AR and PPAR*γ* signaling pathways are clinically relevant in light of the fact that AR ligands are used to treat hormone imbalances and various diseases of the prostate. AR antagonists and compounds that indirectly reduce AR function by altering the level of circulating androgens are routinely used to treat early and late stage prostate cancer as well as a second prostatic disease, benign prostatic hyperplasia. Injections of the AR agonist testosterone are used to treat male patients with delayed puberty or impotence. The PPAR*γ* agonists rosiglitazone and pioglitazone were routinely prescribed for the treatment of type II diabetes during the early to mid-2000s. However, worldwide clinical use of these drugs in recent years has been curtailed due to concerns regarding drug safety. Rosiglitazone was pulled from the European market in 2010 and is now used rarely in the United States due to reports that rosiglitazone enhances the risk of cardiovascular events. Use of the PPAR*γ* agonist pioglitazone has been also suspended in France and Germany due to concerns it may increase one's risk of bladder cancer. However, pioglitazone is still prescribed in the United States as a treatment for type II diabetes in patients without risk factors or a history of bladder cancer. It is therefore possible that AR expression and function are altered within the prostates of diabetic patients taking rosiglitazone or pioglitazone to manage their disease. As new AR and PPAR*γ* ligands are developed for clinical use, we will need to consider how each compound influences the activity of both AR and PPAR*γ*. In conclusion, the research published to date clearly indicates there is bidirectional crosstalk between the PPAR*γ* and AR signaling pathways in human prostate. Additional studies should be conducted to fully understand the significance of this crosstalk in the biology of the normal prostate and other prostatic diseases.

## Figures and Tables

**Table 1:** AR-FL and PPAR*γ* expression within human prostate cancer cell lines.

Cell Line	Castration status	AR-FL Levels	PPAR*γ* levels	Refs.
C4-2	Castration-resistant	++	++	[84, 111]
DU145	Castration-resistant	−	++	[82]
LNCaP	Castration-sensitive	+	+	[82, 84]
PC3	Castration-resistant	−	+++	[82, 84]
VCaP	Castration-sensitive	++	+	[111]

^−^Little to no expression; ^+^low expression; ^++^intermediate expression; and ^+++^high expression.

## Abbreviations

ADT: Androgen deprivation therapy
AR: Androgen receptor
ARE: Androgen response elements
AR-FL: Full-length androgen receptor
ARVs: Androgen receptor variants
CD1: Cyclin D1
DBD: DNA binding domain
DHT: Dihydrotestosterone
FABPs: Fatty acid binding proteins
FABP4: Adipocyte fatty acid binding protein
FABP5: Cutaneous fatty acid binding protein
FGFs: Fibroblast growth factors
IGF-I: Insulin-like growth factor I
LBD: Ligand binding domain
miRNAs: MicroRNAs
NCCN: National Comprehensive Cancer Network
NCoR: Nuclear corepressor
NTD: N terminal domain
PGC-1*α*: Peroxisome proliferator activated receptor coactivator 1 alpha
PIN: Prostatic intraepithelial neoplasia
PPAR*γ*: Peroxisome proliferator activated receptor gamma
PPREs: PPAR response elements
PSA: Prostate-specific antigen
RXR: Retinoid X receptor
SRC-1: Steroid receptor coactivator-1
TIF-2: Transcriptional intermediary factor-2
TRAP: Transgenic rat for adenocarcinoma of prostate
TZDs: Thiazolidinediones
UGE: Urogenital sinus epithelium
UGM: Urogenital sinus mesenchyme
VEGF: Vascular endothelial growth factor
15dPGJ2: 15-Deoxy-Δ^12,14^-prostaglandin J2
15S-HETE: 15(S)-Hydroxyeicosatetraenoic acid.

## References

[1] Watt K. W., Lee P. J., M'Timkulu T., Chan W. P., Loor R. (1986). Human prostate-specific antigen: structural and functional similarity with serine proteases.. *Proceedings of the National Acadamy of Sciences of the United States of America*.

[2] Wang M., Valenzuela L., Murphy G., Chu T. (1979). Purification of a human prostate specific antigen. *Investigative Urology*.

[3] Lilja H., Oldbring J., Rannevik G., Laurell C. B. (1987). Seminal vesicle-secreted proteins and their reactions during gelation and liquefaction of human semen.. *The Journal of Clinical Investigation*.

[4] A.C. Society Cancer facts & figures.

[5] Castro E., Eeles R. (2012). The role of BRCA1 and BRCA2 in prostate cancer. *Asian Journal of Andrology*.

[6] Ryan S., Jenkins M. A., Win A. K. (2014). Risk of prostate cancer in lynch syndrome: A systematic review and meta-Analysis. *Cancer Epidemiology, Biomarkers & Prevention*.

[7] Cao Y., Giovannucci E. (2016). Obesity and prostate cancer. *Recent Results in Cancer Research*.

[8] Vidal A. C., Howard L. E., Moreira D. M., Castro-Santamaria R., Andriole G. L., Freedland S. J. (2014). Obesity increases the risk for high-grade prostate cancer: Results from the REDUCE study. *Cancer Epidemiology, Biomarkers & Prevention*.

[9] Wu V. J., Pang D., Tang W. W., Zhang X., Li L., You Z. (2017). Obesity, age, ethnicity, and clinical features of prostate cancer patients. *American Journal of Clinical and Experimental Urology*.

[10] N.C.C. Network Nccn clinical practice guidelines in oncololgy: Prostate cancer (version 2.2017). https://www.nccn.org/professionals/physician_gls/pdf/prostate.pdf.

[11] Lonergan P. E., Tindall D. J. (2011). Androgen receptor signaling in prostate cancer development and progression. *Journal of Carcinogenesis*.

[12] Wahli W., Martinez E. (1991). Superfamily of steroid nuclear receptors: positive and negative regulators of gene expression. *The FASEB Journal*.

[13] Chang C. S., Kokontis J., Liao S. (1988). Molecular cloning of human and rat complementary DNA encoding androgen receptors. *Science*.

[14] Lubahn D. B., Joseph D. R., Sullivan P. M., Willard H. F., French F. S., Wilson E. M. (1988). Cloning of human androgen receptor complementary DNA and localization to the X chromosome. *Science*.

[15] Liao S., Kokontis J., Sai T., Hiipakka R. A. (1989). Androgen receptors: Structures, mutations, antibodies and cellular dynamics. *The Journal of Steroid Biochemistry and Molecular Biology*.

[16] Smith D. F., Toft D. O. (1993). Steroid receptors and their associated proteins. *Molecular Endocrinology*.

[17] Georget V., Lobaccaro J. M., Terouanne B., Mangeat P., Nicolas J.-C., Sultan C. (1997). Trafficking of the androgen receptor in living cells with fused green fluorescent protein-androgen receptor. *Molecular and Cellular Endocrinology*.

[18] Tyagi R. K., Lavrovsky Y., Ahn S. C., Song C. S., Chatterjee B., Roy A. K. (2000). Dynamics of intracellular movement and nucleocytoplasmic recycling of the ligand-activated androgen receptor in living cells. *Molecular Endocrinology*.

[19] Ruizeveld De Winter J. A., Trapman J., Vermey M., Mulder E., Zegers N. D., Van der Kwast T. H. (1991). Androgen receptor expression in human tissues: An immunohistochemical study. *Journal of Histochemistry & Cytochemistry*.

[20] Tuxhorn J. A., Ayala G. E., Rowley D. R. (2001). Reactive stroma in prostate cancer progression. *The Journal of Urology*.

[21] Hayward S., Rosen M., Cunha G. (1997). Stromal-epithelial interactions in the normal and neoplastic prostate. *British Journal of Urology*.

[22] Cunha G. R., Hayward S. W., Wang Y. Z., Ricke W. A. (2003). Role of the stromal microenvironment in carcinogenesis of the prostate. *International Journal of Cancer*.

[23] Cunha G. R. (1994). Role of mesenchymal‐epithelial interactions in normal and abnormal development of the mammary gland and prostate. *Cancer*.

[24] Hayward S. W., Cunha G. R. (2000). The prostate: Development and physiology. *Radiologic Clinics of North America*.

[25] Evangelou A. I., Winter S. F., Huss W. J., Bok R. A., Greenberg N. M. (2004). Steroid hormones, polypeptide growth factors, hormone refractory prostate cancer, and the neuroendocrine phenotype. *Journal of Cellular Biochemistry*.

[26] Wang Y., Hayward S. W., Cao M., Thayer K. A., Cunha G. R. (2001). Cell differentiation lineage in the prostate. *Differentiation*.

[27] Cunha G. R., Lung B. (1978). The possible influence of temporal factors in androgenic responsiveness of urogenital tissue recombinants from wild‐type and androgen‐insensitive (Tfm) Mice. *Journal of Experimental Zoology*.

[28] Yu S., Zhang C., Lin C.-C. (2011). Altered prostate epithelial development and IGF-1 signal in mice lacking the androgen receptor in stromal smooth muscle cells. *The Prostate*.

[29] Yu S., Yeh C.-R., Niu Y. (2012). Altered prostate epithelial development in mice lacking the androgen receptor in stromal fibroblasts. *The Prostate*.

[30] Bonkhoff H., Remberger K. (1993). Widespread distribution of nuclear androgen receptors in the basal cell layer of the normal and hyperplastic human prostate. *Virchows Archiv A Pathological Anatomy and Histopathology*.

[31] Prins G. S., Birch L., Greene G. L. (1991). Androgen receptor localization in different cell types of the adult rat prostate. *Endocrinology*.

[32] Evans G. S., Chandler J. A. (1987). Cell proliferation studies in rat prostate I. the proliferative role of basal and secretory epithelial cells during normal growth. *The Prostate*.

[33] Cunha G. R., Bigsby R. M., Cooke P. S., Sugimura Y. (1985). Stromal-epithelial interactions in adult organs. *Cell Differentiation*.

[34] Donjacour A. A., Cunha G. R. (1988). The effect of androgen deprivation on branching morphogenesis in the mouse prostate. *Developmental Biology*.

[35] Kwabi-Addo B., Ozen M., Ittmann M. (2004). The role of fibroblast growth factors and their receptors in prostate cancer. *Endocrine-Related Cancer*.

[36] Levine A. C., Liu X.-H., Greenberg P. D. (1998). Androgens induce the expression of vascular endothelial growth factor in human fetal prostatic fibroblasts. *Endocrinology*.

[37] Lai K.-P., Yamashita S., Vitkus S., Shyr C.-R., Yeh S., Chang C. (2012). Suppressed prostate epithelial development with impaired branching morphogenesis in mice lacking stromal fibromuscular androgen receptor. *Molecular Endocrinology*.

[38] Arnold J. T., Isaacs J. T. (2002). Mechanisms involved in the progression of androgen-independent prostate cancers: It is not only the cancer cell's fault. *Endocrine-Related Cancer*.

[39] Wong Y., Wang Y. (2000). Growth factors and epithelial-stromal interactions in prostate cancer development.

[40] Ohlson N., Bergh A., Stattin P., Wikström P. (2007). Castration-induced epithelial cell death in human prostate tissue is related to locally reduced IGF-1 levels. *The Prostate*.

[41] Hayward S. W., Hom Y. K., Grossfeld G. D. (2001). Malignant transformation in a nontumorigenic human prostatic epithelial cell line. *Cancer Research*.

[42] Ricke E. A., Williams K., Lee Y.-F. (2012). Androgen hormone action in prostatic carcinogenesis: Stromal androgen receptors mediate prostate cancer progression, malignant transformation and metastasis. *Carcinogenesis*.

[43] Leach D. A., Buchanan G. (2017). Stromal androgen receptor in prostate cancer development and progression. *Cancers*.

[44] Lapouge G., Erdmann E., Marcias G. (2007). Unexpected paracrine action of prostate cancer cells harboring a new class of androgen receptor mutation - A new paradigm for cooperation among prostate tumor cells. *International Journal of Cancer*.

[45] Dehm S. M., Tindall D. J. (2011). Alternatively spliced androgen receptor variants. *Endocrine-Related Cancer*.

[46] Wadosky K. M., Koochekpour S. (2017). Androgen receptor splice variants and prostate cancer: From bench to bedside. *Oncotarget *.

[47] Ware K. E., Garcia-Blanco M. A., Armstrong A. J., Dehm S. M. (2014). Biologic and clinical significance of androgen receptor variants in castration resistant prostate cancer. *Endocrine-Related Cancer*.

[48] Dehm S. M., Schmidt L. J., Heemers H. V., Vessella R. L., Tindall D. J. (2008). Splicing of a novel androgen receptor exon generates a constitutively active androgen receptor that mediates prostate cancer therapy resistance. *Cancer Research*.

[49] Marcias G., Erdmann E., Lapouge G. (2010). Identification of novel truncated Androgen Receptor (AR) mutants including unreported pre-mRNA splicing variants in the 22Rv1 hormone-refractory Prostate Cancer (PCa) cell line. *Human Mutation*.

[50] Guo Z., Yang X., Sun F. (2009). A novel androgen receptor splice variant is up-regulated during prostate cancer progression and promotes androgen depletion-resistant growth. *Cancer Research*.

[51] Hu R., Isaacs W. B., Luo J. (2011). A snapshot of the expression signature of androgen receptor splicing variants and their distinctive transcriptional activities. *The Prostate*.

[52] Watson P. A., Chen Y. F., Balbas M. D. (2010). Constitutively active androgen receptor splice variants expressed in castration-resistant prostate cancer require full-length androgen receptor. *Proceedings of the National Acadamy of Sciences of the United States of America*.

[53] Hu R., Dunn T. A., Wei S. (2009). Ligand-independent androgen receptor variants derived from splicing of cryptic exons signify hormone-refractory prostate cancer. *Cancer Research*.

[54] Hörnberg E., Ylitalo E. B., Crnalic S. (2011). Expression of androgen receptor splice variants in prostate cancer bone metastases is associated with castration-resistance and short survival. *PLoS ONE*.

[55] Antonarakis E. S., Lu C., Luber B. (2015). Androgen receptor splice variant 7 and efficacy of taxane chemotherapy in patients with metastatic castration-resistant prostate cancer. *JAMA Oncology*.

[56] Antonarakis E. S., Lu C., Luber B., Wang H., Chen Y., Zhu Y. (2017). Clinical significance of androgen receptor splice variant-7 mrna detection in circulating tumor cells of men with metastatic castration-resistant prostate cancer treated with first- and second-line abiraterone and enzalutamide. *Journal of Clinical Oncology*.

[57] Kohli M., Ho Y., Hillman D. W. (2017). Androgen receptor variant AR-V9 is coexpressed with AR-V7 in prostate cancer metastases and predicts abiraterone resistance. *Clinical Cancer Research*.

[58] Xu D., Zhan Y., Qi Y. (2015). Androgen receptor splice variants dimerize to transactivate target genes. *Cancer Research*.

[59] Feldman B. J., Feldman D. (2001). The development of androgen-independent prostate cancer. *Nature Reviews Cancer*.

[60] Sun S., Sprenger C. C. T., Vessella R. L. (2010). Castration resistance in human prostate cancer is conferred by a frequently occurring androgen receptor splice variant. *The Journal of Clinical Investigation*.

[61] Huggins C., Hodges C. V. (2002). Studies on prostatic cancer: I. The effect of castration, of estrogen and of androgen injection on serum phosphatases in metastatic carcinoma of the prostate. 1941.. *The Journal of Urology*.

[62] Lee K. L., Peehl D. M. (2004). Molecular and cellular pathogenesis of benign prostatic hyperplasia. *The Journal of Urology*.

[63] Santen R. J. (1992). Clinical review 37: Endocrine treatment of prostate cancer. *The Journal of Clinical Endocrinology & Metabolism*.

[64] Knudsen K. E., Penning T. M. (2010). Partners in crime: Deregulation of AR activity and androgen synthesis in prostate cancer. *Trends in Endocrinology & Metabolism*.

[65] Knudsen K. E., Scher H. I. (2009). Starving the addiction: New opportunities for durable suppression of AR signaling in prostate cancer. *Clinical Cancer Research*.

[66] Lamont K. R., Tindall D. J. (2011). Minireview: Alternative activation pathways for the androgen receptor in prostate cancer. *Molecular Endocrinology*.

[67] Ho Y., Dehm S. M. (2017). Androgen receptor rearrangement and splicing variants in resistance to endocrine therapies in prostate cancer. *Endocrinology*.

[68] McCrea E., Sissung T. M., Price D. K., Chau C. H., Figg W. D. (2016). Androgen receptor variation affects prostate cancer progression and drug resistance. *Pharmacological Research*.

[69] Dalal K., Roshan-Moniri M., Sharma A. (2014). Selectively targeting the DNA-binding domain of the androgen receptor as a prospective therapy for prostate cancer. *The Journal of Biological Chemistry*.

[70] Tontonoz P., Spiegelman B. M. (2008). Fat and beyond: the diverse biology of PPAR*γ*. *Annual Review of Biochemistry*.

[71] Jiang M., Shappell S. B., Hayward S. W. (2004). Approaches to understanding the importance and clinical implications of peroxisome proliferator-activated receptor gamma (PPAR*γ*) signaling in prostate cancer. *Journal of Cellular Biochemistry*.

[72] Diezko R., Suske G. (2013). Ligand binding reduces sumoylation of the peroxisome proliferator-activated receptor *γ* (PPAR*γ*) activation function 1 (AF1) domain. *PLoS ONE*.

[73] Ohshima T., Koga H., Shimotahno K. (2004). Transcriptional activity of peroxisome proliferator-activated receptor *γ* is modulated by SUMO-1 modification. *The Journal of Biological Chemistry*.

[74] Pascual G., Fong A. L., Ogawa S. (2005). A SUMOylation-dependent pathway mediates transrepression of inflammatory response genes by PPAR-*γ*. *Nature*.

[75] Grøntved L., Madsen M. S., Boergesen M., Roeder R. G., Mandrup S. (2010). MED14 tethers mediator to the N-terminal domain of peroxisome proliferator-activated receptor *γ* and is required for full transcriptional activity and adipogenesis. *Molecular and Cellular Biology*.

[76] Fajas L., Auboeuf D., Raspé E. (1997). The organization, promoter analysis, and expression of the human PPAR*γ* gene. *The Journal of Biological Chemistry*.

[77] Meirhaeghe A., Fajas L., Gouilleux F. (2003). A functional polymorphism in a STAT5B site of the human PPAR*γ*3 gene promoter affects height and lipid metabolism in a French population. *Arteriosclerosis, Thrombosis, and Vascular Biology*.

[78] Chaffer C. L., Thomas D. M., Thompson E. W., Williams E. D. (2006). PPAR*γ*-independent induction of growth arrest and apoptosis in prostate and bladder carcinoma. *BMC Cancer*.

[79] Subbarayan V., Sabichi A. L., Kim J. (2004). Differential peroxisome proliferator-activated receptor-*γ* isoform expression and agonist effects in normal and malignant prostate cells. *Cancer Epidemiology, Biomarkers & Prevention*.

[80] Jiang M., Fernandez S., Jerome W. G. (2010). Disruption of PPAR*γ* signaling results in mouse prostatic intraepithelial neoplasia involving active autophagy. *Cell Death & Differentiation*.

[81] Ahmad I., Mui E., Galbraith L. (2016). Sleeping beauty screen reveals Pparg activation in metastatic prostate cancer. *Proceedings of the National Acadamy of Sciences of the United States of America*.

[82] Mueller E., Smith M., Sarraf P. (2000). Effects of ligand activation of peroxisome proliferator-activated receptor *γ* in human prostate cancer. *Proceedings of the National Acadamy of Sciences of the United States of America*.

[83] Segawa Y., Yoshimura R., Hase T. (2002). Expression of peroxisome proliferator-activated receptor (PPAR) in human prostate cancer. *The Prostate*.

[84] Moss P. E., Lyles B. E., Stewart L. V. (2010). The PPAR*γ* ligand ciglitazone regulates androgen receptor activation differently in androgen-dependent versus androgen-independent human prostate cancer cells. *Experimental Cell Research*.

[85] Hsi L. C., Wilson L. C., Eling T. E. (2002). Opposing effects of 15-lipoxygenase-1 and -2 metabolites on MAPK signaling in prostate: Alteration in peroxisome proliferator-activated receptor *γ*. *The Journal of Biological Chemistry*.

[86] Shappell S. B., Gupta R. A., Manning S. (2001). 15S-hydroxyeicosatetraenoic acid activates peroxisome proliferator-activated receptor *γ* and inhibits proliferation in PC3 prostate carcinoma cells. *Cancer Research*.

[87] Tang S., Bhatia B., Maldonado C. J. (2002). Evidence that arachidonate 15-lipoxygenase 2 is a negative cell cycle regulator in normal prostate epithelial cells. *The Journal of Biological Chemistry*.

[88] Akinyeke T. O., Stewart L. V. (2011). Troglitazone suppresses c-Myc levels in human prostate cancer cells via a PPAR*γ*-independent mechanism. *Cancer Biology & Therapy*.

[89] Kubota T., Koshizuka K., Williamson E. A. (1998). Ligand for peroxisome proliferator-activated receptor gamma (Troglitazone) has potent antitumor effect against human prostate cancer both in vitro and in vivo. *Cancer Research*.

[90] Lyles B. E., Akinyeke T. O., Moss P. E., Stewart L. V. (2009). Thiazolidinediones regulate expression of cell cycle proteins in human prostate cancer cells via PPAR*γ*-dependent and PPAR*γ*-independent pathways. *Cell Cycle*.

[91] Panigrahy D., Singer S., Shen L. Q. (2002). PPAR*γ* ligands inhibit primary tumor growth and metastasis by inhibiting angiogenesis. *The Journal of Clinical Investigation*.

[92] Xu Y., Iyengar S., Roberts R. L., Shappell S. B., Peehl D. M. (2003). Primary culture model of peroxisome proliferator-activated receptor *γ* activity in prostate cancer cells. *Journal of Cellular Physiology*.

[93] Suzuki S., Mori Y., Nagano A. (2016). Pioglitazone, a peroxisome proliferator-activated receptor *γ* agonist, suppresses rat prostate carcinogenesis. *International Journal of Molecular Sciences*.

[94] Wei S., Lin L.-F., Yang C.-C. (2007). Thiazolidinediones modulate the expression of *β*-catenin and other cell-cycle regulatory proteins by targeting the F-box proteins of Skp1-Cul1-F-box protein E3 ubiquitin ligase independently of peroxisome proliferator-activated receptor *γ*. *Molecular Pharmacology*.

[95] Santha S., Davaakhuu G., Basu A. (2016). Modulation of glycogen synthase kinase-3*β* following TRAIL combinatorial treatment in cancer cells. *Oncotarget *.

[96] Wang C., Fu M., D'Amico M., Albanese C., Zhou J. N., Brownlee M. (2001). Inhibition of cellular proliferation through ikappab kinase-independent and peroxisome proliferator-activated receptor gamma-dependent repression of cyclin d1. *Molecular & Cellular Biology*.

[97] Yin F., Wakino S., Liu Z. (2001). Troglitazone inhibits growth of MCF-7 breast carcinoma cells by targeting G1 cell cycle regulators. *Biochemical and Biophysical Research Communications*.

[98] Qin C., Burghardt R., Smith R., Wormke M., Stewart J., Safe S. (2003). Peroxisome proliferator-activated receptor *γ* agonists induce proteasome-dependent degradation of cyclin D1 and estrogen receptor *α* in MCF-7 breast cancer cells. *Cancer Research*.

[99] Huang J.-W., Shiau C.-W., Yang Y.-T. (2005). Peroxisome proliferator-activated receptor *γ*-independent ablation of cyclin D1 by thiazolidinediones and their derivatives in breast cancer cells. *Molecular Pharmacology*.

[100] Cerbone A., Toaldo C., Laurora S. (2007). 4-Hydroxynonenal and PPAR*γ* ligands affect proliferation, differentiation, and apoptosis in colon cancer cells. *Free Radical Biology & Medicine*.

[101] Lea M. A., Sura M., Desbordes C. (2004). Inhibition of cell proliferation by potential peroxisome proliferator-activated receptor (PPAR) gamma agonists and antagonists. *Anticancer Reseach*.

[102] Rossi A., Kapahi P., Natoli G. (2000). Anti-inflammatory cyclopentenone prostaglandins are direct inhibitors of I*κ*B kinase. *Nature*.

[103] Strand D. W., Jiang M., Murphy T. A. (2012). PPAR*γ* isoforms differentially regulate metabolic networks to mediate mouse prostatic epithelial differentiation. *Cell Death & Disease*.

[104] Hisatake J. I., Ikezoe T., Carey M., Holden S., Tomoyasu S., Koeffler H. P. (2000). Down-regulation of prostate-specific antigen expression by ligands for peroxisome proliferator-activated receptor *γ* in human prostate cancer. *Cancer Research*.

[105] Yang C.-C., Ku C.-Y., Wei S. (2006). Peroxisome proliferator-activated receptor *γ*-independent repression of prostate-specific antigen expression by thiazolidinediones in prostate cancer cells. *Molecular Pharmacology*.

[106] Yang C.-C., Wang Y.-C., Wei S. (2007). Peroxisome proliferator-activated receptor *γ*-independent suppression of androgen receptor expression by troglitazone mechanism and pharmacologic exploitation. *Cancer Research*.

[107] Nagata D., Yoshihiro H., Nakanishi M. (2008). Peroxisome proliferator-activated receptor-*γ* and growth inhibition by its ligands in prostate cancer. *Cancer Epidemiology*.

[108] Kaikkonen S., Paakinaho V., Sutinen P., Levonen A.-L., Palvimo J. J. (2013). Prostaglandin 15d-PGJ2 inhibits androgen receptor signaling in prostate cancer cells. *Molecular Endocrinology*.

[109] Tew B. Y., Hong T. B., Otto-Duessel M. (2017). Vitamin K epoxide reductase regulation of androgen receptor activity. *Oncotarget *.

[110] Seargent J. M., Yates E. A., Gill J. H. (2004). GW9662, a potent antagonist of PPAR*γ*, inhibits growth of breast tumour cells and promotes the anticancer effects of the PPAR*γ* agonist rosiglitazone, independently of PPAR*γ* activation. *British Journal of Pharmacology*.

[111] Olokpa E., Bolden A., Stewart L. V. (2016). The Androgen Receptor Regulates PPAR*γ* Expression and Activity in Human Prostate Cancer Cells. *Journal of Cellular Physiology*.

[112] Singh R., Artaza J. N., Taylor W. E. (2006). Testosterone inhibits adipogenic differentiation in 3T3-L1 cells: nuclear translocation of androgen receptor complex with *β*-catenin and T-cell factor 4 may bypass canonical Wnt signaling to down-regulate adipogenic transcription factors. *Endocrinology*.

[113] Lefebvre A.-M., Peinado-Onsurbe J., Leitersdorf I. (1997). Regulation of lipoprotein metabolism by thiazolidinediones occurs through a distinct but complementary mechanism relative to fibrates. *Arteriosclerosis, Thrombosis, and Vascular Biology*.

[114] Noy N., Morgan E., Kannan-Thulasiraman P. (2010). Involvement of fatty acid binding protein 5 and PPAR *β*/*δ* in prostate cancer cell growth. *PPAR Research*.

[115] De Santis M. L., Hammamieh R., Das R., Jett M. (2004). Adipocyte-fatty acid binding protein induces apoptosis in du145 prostate cancer cells. *Journal of Experimental Therapeutics and Oncology*.

[116] White M. A., Lin C., Rajapakshe K. (2017). Glutamine Transporters Are Targets of Multiple Oncogenic Signaling Pathways in Prostate Cancer. *Molecular Cancer Research*.

[117] Massie C. E., Lynch A., Ramos-Montoya A. (2011). The androgen receptor fuels prostate cancer by regulating central metabolism and biosynthesis. *EMBO Journal*.

[118] Puigserver P., Wu Z., Park C. W., Graves R., Wright M., Spiegelman B. M. (1998). A cold-inducible coactivator of nuclear receptors linked to adaptive thermogenesis. *Cell*.

[119] Shiota M., Yokomizo A., Tada Y. (2010). Peroxisome proliferator-activated receptor *γ* coactivator-1*α* interacts with the androgen receptor (AR) and promotes prostate cancer cell growth by activating the AR. *Molecular Endocrinology*.

[120] Tennakoon J. B., Shi Y., Han J. J. (2014). Androgens regulate prostate cancer cell growth via an AMPK-PGC-1*α*-mediated metabolic switch. *Oncogene*.

[121] Oñate S. A., Tsai S. Y., Tsai M.-J., O'Malley B. W. (1995). Sequence and characterization of a coactivator for the steroid hormone receptor superfamily. *Science*.

[122] Voegel J. J., Heine M. J. S., Zechel C., Chambon P., Gronemeyer H. (1996). TIF2, a 160 kDa transcriptional mediator for the ligand-dependent activation function AF-2 of nuclear receptors. *EMBO Journal*.

[123] Ogryzko V. V., Schiltz R. L., Russanova V., Howard B. H., Nakatani Y. (1996). The transcriptional coactivators p300 and CBP are histone acetyltransferases. *Cell*.

[124] Bannister A. J., Kouzarides T. (1996). The CBP co-activator is a histone acetyltransferase. *Nature*.

[125] Agoulnik I. U., Vaid A., Bingman W. E. (2005). Role of SRC-1 in the promotion of prostate cancer cell growth and tumor progression. *Cancer Research*.

[126] Gregory C. W., He B., Johnson R. T. (2001). A mechanism for androgen receptor-mediated prostate cancer recurrence after androgen deprivation therapy. *Cancer Research*.

[127] Knudsen K. E., Cavenee W. K., Arden K. C. (1999). D-type cyclins complex with the androgen receptor and inhibit its transcriptional transactivation ability. *Cancer Research*.

[128] Petre C. E., Wetherill Y. B., Danielsen M., Knudsen K. E. (2002). Cyclin D1: Mechanism and consequence of androgen receptor co-repressor activity. *The Journal of Biological Chemistry*.

[129] Berrevoets C. A., Umar A., Trapman J., Brinkmann A. O. (2004). Differential modulation of androgen receptor transcriptional activity by the nuclear receptor co-repressor (N-CoR). *Biochemical Journal*.

[130] Hodgson M. C., Astapova I., Cheng S. (2005). The androgen receptor recruits nuclear receptor corepressor (N-CoR) in the presence of mifepristone via its N and C termini revealing a novel molecular mechanism for androgen receptor antagonists. *The Journal of Biological Chemistry*.

[131] Li P., Fan W., Xu J. (2011). Adipocyte NCoR knockout decreases PPAR*γ* phosphorylation and enhances PPAR*γ* activity and insulin sensitivity. *Cell*.

[132] Battaglia S., Maguire O., Thorne J. L. (2010). Elevated NCOR1 disrupts PPAR*α*/*γ* signaling in prostate cancer and forms a targetable epigenetic lesion. *Carcinogenesis*.

[133] Kim S. Y., Kim A. Y., Lee H. W. (2010). miR-27a is a negative regulator of adipocyte differentiation via suppressing PPAR*γ* expression. *Biochemical and Biophysical Research Communications*.

[134] Lee J.-J., Drakaki A., Iliopoulos D., Struhl K. (2012). MiR-27b targets PPAR*γ* to inhibit growth, tumor progression and the inflammatory response in neuroblastoma cells. *Oncogene*.

[135] Lee E. K., Lee M. J., Abdelmohsen K. (2011). miR-130 suppresses adipogenesis by inhibiting peroxisome proliferator-activated receptor *γ* expression. *Molecular and Cellular Biology*.

[136] Jeong B.-C., Kang I.-H., Koh J.-T. (2014). MicroRNA-302a inhibits adipogenesis by suppressing peroxisome proliferator-activated receptor *γ* expression. *FEBS Letters*.

[137] Li X., Chen Y., Wu S. (2015). MicroRNA-34a and microRNA-34c promote the activation of human hepatic stellate cells by targeting peroxisome proliferator-activated receptor *γ*. *Molecular Medicine Reports*.

[138] Fletcher C. E., Dart D. A., Sita-lumsden A., Cheng H., Rennie P. S., Bevan C. L. (2012). Androgen-regulated processing of the oncomir MiR-27a, which targets Prohibitin in prostate cancer. *Human Molecular Genetics*.

[139] Östling P., Leivonen S.-K., Aakula A. (2011). Systematic analysis of microRNAs targeting the androgen receptor in prostate cancer cells. *Cancer Research*.

